# Reticulocyte Maturation and Variant Red Blood Cells

**DOI:** 10.3389/fphys.2022.834463

**Published:** 2022-03-07

**Authors:** Christian J. Stevens-Hernandez, Joanna F. Flatt, Sabine Kupzig, Lesley J. Bruce

**Affiliations:** ^1^Bristol Institute for Transfusion Sciences, NHS Blood and Transplant, Bristol, United Kingdom; ^2^School of Biochemistry, University of Bristol, Bristol, United Kingdom; ^3^Component Development Laboratory, NHS Blood and Transplant, Long Road, Cambridge Biomedical Campus, Cambridge, United Kingdom

**Keywords:** reticulocyte maturation, stomatocytosis, OHSt, hereditary spherocytosis, Southeast Asian ovalocytosis, cryohydrocytosis, stomatin, erythropoiesis

## Abstract

The bone marrow produces billions of reticulocytes daily. These reticulocytes mature into red blood cells by reducing their plasma membrane by 20% and ejecting or degrading residual internal organelles, membranes and proteins not required by the mature cell. This process occurs by autophagy, protein degradation and vesiculation but is not well understood. We previously reported that Southeast Asian Ovalocytic RBCs demonstrate incomplete reticulocyte maturation and we have now extended this study to a number of other variant RBCs. By comparing the profile of a pure reticulocyte preparation of cultured red cells with these variant cells, we show that the largest of these cells, the overhydrated hereditary stomatocytosis cells, are the least mature, they barely reduced their plasma membrane and contain large amounts of proteins that should have been reduced or removed. Intermediate sized variant RBCs appear to be more mature but retain some endoplasmic reticulum and residual membrane proteins. We propose that the size and composition of these variant cell types correlate with the different stages of reticulocyte maturation and provide insight into the reticulocyte maturation process.

## Introduction

On average, 200 billion reticulocytes are produced every day in the bone marrow of healthy individuals. These immature red blood cells (RBCs) must go through a process of maturation to form mature RBCs. Immediately post-enucleation reticulocytes are large (120-140fL) and multi-lobular. This stage is known as an R1 reticulocyte as classified by Mel et al., 1977. R1 reticulocytes are motile and are found in the bone marrow; they still contain substantial amounts of RNA giving the cell a reticulo-filamentous appearance and its name. R1 reticulocytes also contain residual mitochondria, ribosomes, endoplasmic reticulum (ER) and other internal membranes that are not required by the mature RBC. They have excess plasma membrane, which must be reduced by 20%, plus numerous superfluous proteins, e.g., the transferrin receptor (TfR) and various integrins, to be removed from the mature RBC (Koury et al., 1989; Wickrema et al., 1994; Liu et al., 2010). This initial stage of maturation occurs by membrane rearrangement via autophagic, proteolytic, and vesicle-based mechanisms (Minetti et al., 2020) and results in the R2 reticulocytes that are released into the circulation where they mature further.

Studies have shown that during maturation the level of RNA, as measured by thiazole orange (TO), and TfR in the reticulocyte reduces and these levels are often used to define the different stages of maturation from R1 (nascent reticulocyte, high CD71, high RNA) to R2 (intermediate reticulocyte, low CD71, low RNA) to R3 (mature RBC, CD71 negative, RNA negative) (Malleret et al., 2013). Recently, another method for classifying different stages of reticulocyte maturation has been described that uses changes in mitochondrial membrane potential (Du et al., 2020). Despite all these classifications, exactly how the nascent reticulocyte matures into a RBC is not fully understood. However, one fact is clear, as the nascent reticulocyte matures it becomes smaller. This is due to the loss of plasma membrane and volume via vesicle release.

The nascent reticulocyte begins life at 120-140fL, then loses membrane forming an R2 reticulocyte of about 100-120fL, further membrane is lost to form a mature RBC of about 86-98fL. This final maturation stage is probably the least understood and must involve the membrane and cytoskeleton “clicking” into their final arrangement, forming a perfect deformable biconcave disk. Interestingly, there are a number of variant RBCs that have mean cell volumes (MCV) of similar sizes to R1 and R2 reticulocytes. These variants cause conditions that affect RBC cation permeability. Overhydrated hereditary stomatocytic (OHSt) RBCs have the most severe cation leak of the HSt group (40x normal) and a MCV of 120-140fL (Gallagher et al., 2003; Bruce et al., 2009). OHSt is caused by variants of *RHAG*, the gene encoding the Rh-associated glycoprotein (RhAG). OHSt RBCs also lack stomatin, although no gene defect was found in *STOM* to account for this loss (Fricke et al., 2003). South-east Asian Ovalocytosis (SAO), cryohydrocytosis (CHC) and stomatin-deficient CHC (sdCHC) have an intermediate cation leak (4-10x normal) and MCVs between 100-120fL. SAO and CHC are caused by variants of *SLC4A1*, the gene encoding band 3 [anion exchanger 1 (AE1)], and have a cation leak of about four times normal (Tanner et al., 1991; Bruce et al., 1999, 2005; Guizouarn et al., 2011). sdCHC is caused by variants of *SLC2A1*, the gene encoding glucose transporter 1 (GLUT1), and these RBCs have a cation leak about ten times normal (Flatt et al., 2011; Bawazir et al., 2012). sdCHC RBCs also lack stomatin although again there is no associated gene defect in *STOM*. We reported previously that SAO RBCs appeared to have a defect in reticulocyte maturation (Flatt et al., 2020). Consequently, we decided to investigate the degree of defective reticulocyte maturation in all of these variant RBCs to determine whether this correlated with their MCV and potentially with the MCV of the different stages of reticulocyte maturation.

## Materials and Methods

### Patient Samples

Control RBC, with no known RBC defects, were isolated from United Kingdom blood donor samples. Variant RBC samples were from an OHSt patient (heterozygous Phe65Ser variant in *RHAG*; rs863225468) first reported by Lock et al., 1961, then again as patient Stockport-A-II-1 (Bruce et al., 2009); a hereditary spherocytosis (HS) patient (heterozygous g > t mutation in the donor splice site of intron 12 of *SLC4A1*; unpublished, see Supplementary Case study); an HS patient with incomplete distal renal tubular acidosis (dRTA) (homozygous Ser667Phe variant in *SLC4A1*), previously reported (Toye et al., 2008); a CHC patient (heterozygous Ser731Pro variant in *SLC4A1*; rs863225461), previously reported as patient CHC2 (Bruce et al., 2005); an sdCHC patient (heterozygous Gly286Asp variant in *SLC2A1*; rs864309514) first reported (Fricke et al., 2004 patient D-II-2, later in Flatt et al., 2011); an SAO sample (heterozygous deletion Ala400-Ala408 in *SLC4A1*; rs769664228) mother of the homozygous SAO child reported (Picard et al., 2014, later in Flatt et al., 2020). Cultured RBCs (cRBCs) were grown *in vitro* from CD34^+^cells isolated from peripheral blood of United Kingdom blood donors.

All blood samples were collected with informed consent, obtained in accordance with the Declaration of Helsinki. This study is part of a larger study approved by the National Health Service National Research Ethics Service South West entitled “*In Vitro* Studies of Erythropoiesis in Health and Disease.”

### Culture of CD34^+^ Cells

CD34^+^ cells were isolated from cones, a by-product of platelet apheresis, and cultured as described (Griffiths et al., 2012a). Typically, a culture begins with ∼2 × 10^6^ CD34^+^cells and expands to ∼5.25 × 10^9^ cells (erythroblasts & reticulocytes) in a 3 liter culture (final volume). Briefly, cells were cultured in IMDM supplemented with 3% (v/v) AB serum (Merck), 2mg/ml HSA (Irvine Scientific), 10 μg/ml insulin (Merck), 3 U/ml Erythropoietin (Roche), 500 μg/ml holotransferrin (R&D Systems), 10 ng/ml SCF (Medsafe), 1 ng/ml IL3 (R&D Systems), 3 IU/ml heparin (Merck) from day 0-10, from day 11-13 as above but without IL-3, then from day 14 onward as for days 11-13 but without SCF. Cultures were kept in vented flasks followed by spinner flasks at 37°C, 5% CO_2_.

### Filtration of Cultured Cells

Cell cultures were filtered, to remove residual nucleated cells and pyrenocytes, using a standard leucofilter (LXT, Macopharma) around day 21, once the enucleated cells were at > 60%. The pure cRBC preparation was resuspended in saline, adenine, glucose and mannitol solution (SAG-M; Macopharma) or SAG-M + 10% human serum albumin (HSA) and stored for 10 days at 4°C.

### Scanning Electron Microscopy

Cells were prepared for scanning electron microscopy as described in Griffiths et al., 2012a. For further details, see Supplementary Methods.

### Erythrocyte Membrane Protein Analysis

RBC ghost membrane preparations were prepared according to the hemolysis method (Dodge et al., 1963), with some modifications (see Supplementary Methods). SDS-PAGE (reducing conditions) and Western blotting analysis were performed as described (Bruce et al., 2003). Blots were analyzed using semi-quantitative scanning densitometry with the Kodak Gel100 system software or LI-COR Image Studio software Densitometry analysis was carried out using Image J (v1.50i) (Supplementary Table 1).

### Antibodies Used for Immunoblotting

Antibodies were used against the following proteins. The voltage dependent anion channel 1 (VDAC1; Abcam, ab15895) and stomatin like protein 2 (SLP2; in-house rabbit polyclonal) were used as markers of residual mitochondrial membranes, calreticulin (Abcam, ab2908) as an endoplasmic reticulum marker, lysosomal associated membrane protein 2 (LAMP-2; Abcam ab25631) as a lysosomal membrane marker and transferrin receptor (TfR; Abcam, ab84036) and CD147 (Abcam, ab108308) as markers of proteins that are significantly reduced during reticulocyte maturation (Gronowicz et al., 1984; Griffiths et al., 2012a; Malleret et al., 2013; Flatt et al., 2020). β-spectrin (BRAC65; IBGRL, Bristol, United Kingdom) was used as a loading control. It was not possible to use β-actin, a common loading control, as β-actin has a similar molecular weight to some of the test proteins. The level of stomatin (STOM, in-house rabbit polyclonal IDML) was assessed because some of the variant RBCs are known to have reduced levels of stomatin (Bruce et al., 2009; Flatt et al., 2011).

### Reprobing Immunoblots

Immunoblots were probed and reprobed in sequence. It was possible to probe for all the proteins of interest, given their different apparent molecular weights, using a limited number of blots. One blot was probed first with anti-VADC1 (35kDa, 37 kDa) then anti-SLP2 (44 kDa). Another blot was probed first with anti-LAMP2 (∼80 kDa) then anti-calreticulin (55 kDa) then anti-STOM (31 kDa). A further blot was probed first with anti-CD147 (30 kDa) (non-glycosylated) and 42-65 kDa (glycosylated) then anti-TfR [98 kDa (monomer), 200 kDa (dimer)]. Between each probing the blot was wetted with methanol, washed with phosphate-buffered saline (PBS; 137mM NaCl, 10mM Phosphate, 2.7mM KCl pH 7.4) Tween solution [PBS-0.2%Tween 20 (Merck)], then re-blocked in 5% milk, PBS-Tween solution and probed. All blots were reprobed with anti-β-spectrin (220 kDa) as a loading control.

## Results

### Production of Normal Human Reticulocytes

**FIGURE 1:**
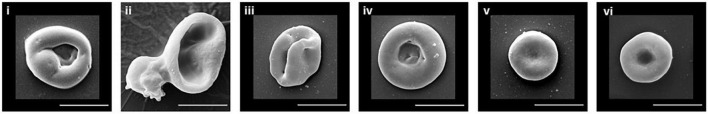
SEM of erythroid cells. Illustrative examples of the typical cell morphology of the different stages of erythrocyte maturation are shown. **(i)** Orthochromatic reticulocyte. **(ii)** Enucleating reticulocyte. **(iii)** R1 reticulocyte. **(iv)** R2 reticulocyte. **(v)** Mature reticulocyte/RBC. **(vi)** Donor RBC. Scale bar 5 μm.

The culture conditions used in our laboratory differentiate the CD34^+^ cells, in a fairly synchronized manner, through to reticulocytes. Using standard blood donor (non-variant) cells we usually achieve 70-80% enucleation and, post-filtration, produce a pure population of cRBCs. Scanning electron micrograph images of the different stages of cell development in culture, from pre-enucleation through to the mature RBC, are shown in Figure 1. Pure cRBCs are a mixture of R1 (Figure 1iii) and R2 (Figure 1iv) reticulocytes plus a small number of more mature cells (Figure 1v) resembling fully mature erythrocytes (Figure 1vi).

### Comparative Mean Cell Volume of Reticulocytes Versus Variant RBCs

The phenotype and properties of the variant RBCs used in this study are shown in Table 1. The mean cell volume (MCV) of standard donor RBCs ranges from 86-98fL whereas reticulocytes have a much larger MCV, ranging from 120-140fL. This reflects the fact that reticulocytes must lose about 20% of their membrane area during the maturation process. RBC variant cells were selected with MCVs intermediate between these two (Table 1). The MCV for the heterozygous SAO sample was in fact lower than the standard range (78fL). MCVs for SAO RBCs in the literature vary enormously and this is because very often SAO is reported in conjunction with other conditions, e.g., thalassemia, where the MCV is low (Yamsri et al., 2021) or distal renal tubular acidosis where the MCV is high (Gunaratne et al., 2020). However, it is clear from heterozygous SAO RBC films that the double stoma, macro-ovalocytes are large (100-120fL; Garnett and Bain, 2013).

**TABLE 1:** RBC properties.

Cell type (sex)	RBC (x10^12^/L)	Hb (g/dL)	MCV fL	MCHC (g/dL)	Retic%	Other complications	References
RBC (normal range)	4.5-5.5 (M) 3.8-4.8 (F)	12.5-18.0 (M) 11.5-15.8 (F)	86-98	30.8-35.3 (adults)	0.5 -1.5 (adults)	N/A	N/A
cRBC	N/A	5.4-6.3	120-140	23.3-37.4	100	N/A	Present study
OHSt (M)	Not reported	9.0-11.0	136.5-139.0	25.3, 27.0	10-20	Compensated hemolytic anemia, stomatocytes, loss of stomatin	Fricke et al., 2003; Bruce et al., 2009
sdCHC (M)	Not reported	12.7	121.3	31.4	1.4	Neurological disorder and cataracts, hemolytic anemia, loss of stomatin	Fricke et al., 2004
CHC (M)	Not reported	15.0	91.2, 87.7	37.3, 38.3	8⋅09, 8⋅99	Mild hemolytic anemia, stomatocytes, gall stones, jaundice.	Coles et al., 1999. (B-III-2)
SAO (F)	5.95^#^	13.9^#^	78^#^	30.2^#^	1.6^#^	Heterozygous 3.7 kb α-thalassemia trait; sickle cell trait (HBB c.20 C > T)	FBC (Picard, unpublished^†^); Picard et al., 2014
HS-het (F)	4.07*	12.2*	90.7*	33*	Not available*	Heterozygous *SLC4A1* variant g > t at c.1431 + 1 affecting the splice donor site of intron 12 and causing HS	Present study (see supplemental data)
HS-hom. (M)	0.94	3.8	100.6	35.9	17.2	Homozygous *SLC4A1* variant (Ser667Phe) causing both HS and dRTA.	Toye et al., 2008

*Full blood counts (FBC) were measured for the cRBC preparations using an automated hematology analyzer (Horiba, United Kingdom). FBC was measured for HS-het patient using hematology analyzer (Sysmex, United Kingdom).*

*^#^The FBC for the SAO was measured in 2011. The MCV for this individual is below the normal range probably due to co-inheritance of the heterozygous 3.7 kb α-thalassemia trait and sickle cell trait (HBB c.20 C > T) (Picard et al., 2014). Indeed, the MCV of heterozygous SAO RBCs often falls within or below the normal range probably due to conditions inherited with SAO. However, SAO RBCs consist of two populations of RBCs, a population of normal sized cells and a population of macro-ovalocytes (100-120fL; Garnett and Bain, 2013). Note, the SAO reticulocyte count is also normal. SAO individuals rarely have reticulocytosis, unless it is associated with other secondary conditions. However neonatal anemia is associated with SAO (Laosombat et al., 2005).*

*The FBC for the HS-het was measured in 2011 post-splenectomy. The reticulocyte count is no longer available but the patient was referred to the Hematology Department with anemia.

*^†^Picard, V., (2022) Personal communication of FBC.*

### Immunoblotting Analysis of Cultured Red Blood Cell Membrane Proteins

**FIGURE 2:**
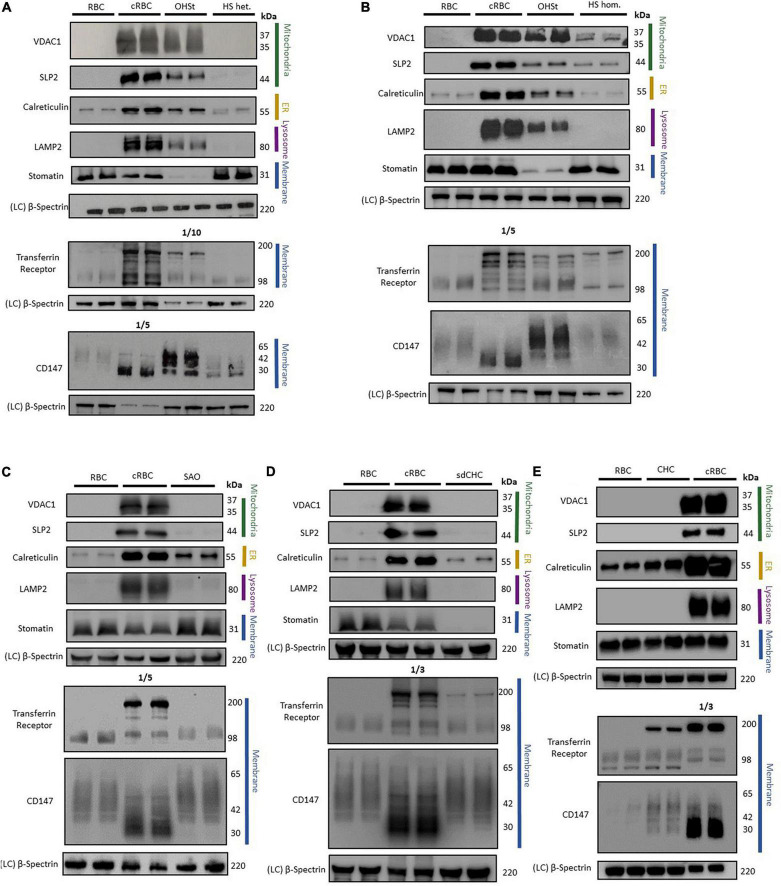
Immunoblotting of RBC membranes. RBC membrane from donor RBCs (RBC) and cultured RBCs (cRBC) were analysed by immunoblotting with **(A)** Overhydrated Hereditary Stomatocytosis RBCs (OHSt) and heterozygous Hereditary Spherocytosis RBCs (HS het.). **(B)** Overhydrated Hereditary Stomatocytosis RBCs (OHSt) and homozygous Hereditary Spherocytosis RBCs (HS hom.). **(C)** Heterozygous South-east Asian Ovalocytosis RBCs (SAO). **(D)** Stomatin-deficient Cryohydrocytosis RBCs (sdCHC). **(E)** Cryohydrocytosis RBCs (CHC). Each immunoblot panel is a representative example of three repeat immunoblots except for panel C (*n* = 1). Densitometry analysis of the immunoblotting data is provided in the supplement.

Cell membranes were isolated from standard donor RBCs and from cRBCs by hypotonic lysis, the proteins separated by SDS-PAGE and analyzed by immunoblotting. As expected, immunoblotting analysis of the cRBC membrane proteins showed large amounts of the proteins associated with mitochondria (VDAC1 & SLP2), with ER (calreticulin) and with lysosomes (LAMP2) compared to vanishingly small amounts of these proteins in the mature donor RBC membranes (Figure 2). Membrane proteins that are usually reduced during reticulocyte maturation (TfR & CD147) were also present in large amounts in the cRBC membranes compared to the donor RBC membranes (Figure 2). The unglycosylated form of CD147 was much increased in the cRBC membranes as was a 200 kDa band in the TfR immunoblot, presumed to be the TfR dimer (Figure 2). Proteins that are known to be present in both reticulocyte and donor RBC membranes (stomatin & β-spectrin) were found to be present in equal amounts in the membranes from both cell types (Figure 2).

### Immunoblotting Analysis of Variant Red Blood Cell Membrane Proteins

Immunoblotting analysis of variant RBC membranes displayed varying levels of the above mentioned proteins, more commonly associated with reticulocytes, suggesting that the variant RBCs may undergo incomplete reticulocyte maturation to different degrees.

#### Overhydrated Hereditary Stomatocytosis Red Blood Cells

Immunoblotting of OHSt RBC membranes gave a profile more similar to that of the cRBC membranes than mature RBC membranes (Figures 2A,B). The OHSt membranes contained VDAC1, SLP2, calreticulin and LAMP2, at about 12-94% the level found in cRBC membranes (Supplementary Table 1) showing that the OHSt RBCs have incomplete clearance of mitochondrial, ER and lysosomes. There was also incomplete clearance of the proteins that are usually reduced during reticulocyte maturation, CD147 and TfR (note that the TfR OHSt blot in Figure 2A is a 1:10 dilution); and the unglycosylated form of CD147 and dimeric form of TfR were both increased (Figures 2A,B). OHSt RBC membranes contained normal levels of spectrin but very low levels of stomatin as expected (Figures 2A,B). The same high level of these proteins (VDAC1, SLP2, calreticulin, LAMP2, CD147 & TfR) was found in a second OHSt sample, confirming that this profile is typical of the OHSt phenotype (data not shown).

#### Hereditary Spherocytosis Red Blood Cells

In contrast, immunoblotting of heterozygous HS RBC membranes gave a similar profile to that of control donor RBC membranes, clearing most internal membranes and containing only residual amounts of calreticulin (ER), TfR and CD147 (Figure 2A and Supplementary Table 1), about the same level as found in the mature RBC membranes, suggesting that HS RBCs complete reticulocyte maturation normally and that the RBC defect occurs later during RBC circulation. Homozygous HS RBC membranes did contain slightly more residual mitochondrial membrane proteins (VDAC1 and SLP2) than donor RBC membranes (Figure 2B and Supplementary Table 1) but this sample has a homozygous *SLC4A1* variant causing low SLC4A1 expression, a trafficking defect and hemolytic anemia (Toye et al., 2008). Both heterozygous and homozygous HS RBC membranes contained normal levels of spectrin and stomatin (Figures 2A,B).

#### South-East Asian Ovalocytosis, Stomatin-Deficient Cryohydrocytosis and Cryohydrocytosis

As reported previously (Flatt et al., 2020), SAO RBCs failed to clear calreticulin as efficiently as control donor RBCs, suggesting greater retention of ER membranes, although mitochondrial membrane proteins (VDAC1 and SLP2) and lysosomal membrane protein (LAMP2) appeared to clear normally (Figure 2C and Supplementary Table 1). CD147 was not cleared as well from SAO membranes as from the control donor RBC membranes and migrated more slowly in SDS-PAGE, whereas TfR was cleared slightly better from SAO compared to control RBC membranes (Figure 2C and Supplementary Table 1). Stomatin and spectrin were present in normal amounts in SAO membranes (Figure 2C).

The sdCHC and CHC RBC membranes gave a similar profile to that of the SAO RBC membranes. Both cleared residual mitochondrial and lysosomal membranes to the same degree as the mature donor RBCs but retained more ER membranes than the donor RBCs (Figures 2D,E and Supplementary Table 1). Both had increased amounts of the monomeric and dimeric forms of TfR relative to the donor RBC membranes (Figures 2D,E). CD147 was increased in the CHC compared to donor RBCs (Figures 2D,E and Supplementary Table 1). Both contained normal levels of spectrin but, as expected, stomatin was reduced in the sdCHC RBCs (Figures 2D,E and Supplementary Table 1).

## Discussion

The culture conditions used in our laboratory differentiate CD34^+^ cells, in a fairly synchronized manner, through to reticulocytes. The ultimate goal is to mature these cells *in vitro* into erythrocytes but, as far as we know, no one has managed to achieve this yet. Native reticulocytes, isolated from peripheral blood, can be matured *in vitro* (Koury et al., 2005). Indeed, we have also shown that native reticulocytes, when incubated in either plasma or media at 37°C for ten days, reduce their levels of RNA and TfR and develop an increased ratio of R2:R1 reticulocytes (data not shown). Cultured reticulocytes can be induced to begin the maturation process when put under shear stress (Moura et al., 2019) and appear to mature fully in the circulation of a mouse model (Kupzig et al., 2017) but completing the maturation process *in vitro* has yet to be achieved. Proteomic analysis comparing native reticulocytes to cultured reticulocytes shows their proteomic profile to be very similar (Moura et al., 2018) but there must be an essential factor missing from the cRBCs (perhaps a plasma protein or the lack of macrophages) that restricts their development *in vitro*. There is a great deal of interest in understanding the mechanism of reticulocyte maturation. Numerous laboratories worldwide are growing cRBCs and these cells have the potential for many future applications, e.g. in drug delivery or therapeutics or even as a future blood component if scale-up and cost-reduction are achieved. However, storage of cRBCs is a problem, as cRBCs are much less stable than mature erythrocytes in cold-storage, but if cRBCs could be matured to erythrocytes then their full potential could be realized.

The process of reticulocyte maturation involves a reduction in size and clearance of residual organelles, internal membranes and proteins that are superfluous to the function of the mature RBC. There have been a number of cellular mechanisms identified. Autophagic organelle clearance is thought to begin pre-enucleation and continue through reticulocyte maturation. Autophagosomes deliver cytoplasmic proteins or organelles to lysosomes for degradation (Mei et al., 2021). This involves the autophagy proteins LC3, ATG4 and ATG7 (Zhang et al., 2009; Betin et al., 2013). Mitophagy has been shown to be inhibited in ATG7-deficient erythroid cells however removal of ribosomes and endoplasmic reticulum continues in the absence of ATG7 and may involve a different mechanism (Zhang et al., 2009). Indeed, ubiquitin proteasome-mediated proteolysis degrades ribosomal proteins as well as other non-ribosomal targets, such as histone H2B and may be the mechanism used for degradation of much of the obsolete cytoplasmic proteins (Nguyen et al., 2017). Removal or reduction of membranes proteins involves endocytosis. Following endocytosis, the endosome invaginates to form exosomes, containing the obsolete membranes proteins, and either fuses with the plasma membrane, discharging its content, or fuses with a lysosome where the proteins are digested.

Levels of CD71 and RNA/reticulin gradually reduce throughout the maturation process and this clearance may involve all of the above mechanisms. However, reduction of TfR from the circulating R2 reticulocyte appears to require the interaction of splenic macrophages (Rhodes et al., 2016) and is probably separate from the mitochondrial clearance stage (Zhang et al., 2019). In late stages of maturation, the final organelle and membrane remnants, together with residual obsolete proteins, are collected in large vacuolar compartments that comprise part endosome that label strongly with glycophorin A (GPA) and part autophagosome that label with LC3 (Griffiths et al., 2012b). These autophagic-endocytic macro-vesicles then squeeze out through the plasma membrane, by a mechanism yet to be determined, and are removed by phagocytes (Mankelow et al., 2016). These large macro-vesicles can be seen inside reticulocytes and partially protruding from reticulocytes and RBCs, in splenectomized individuals, and label positively for phosphatidylserine, a phospholipid usually restricted to the inner membrane leaflet (Mankelow et al., 2016).

### Variant Cells

In this study we have compared the degree of defective reticulocyte maturation in the variant RBCs as measured by their size (MCV) and the levels of proteins, that should have been cleared by the maturation process, remaining in their membranes. We hypothesize that these attributes correlate with the size and reticulocyte-like protein patterns of the different stages of reticulocyte maturation. As expected, post-enucleation cRBC membranes contain large amounts of residual internal membranes, e.g., mitochondria, ER and lysosomes as can be seen by the presence of VDAC1, SLP2, calreticulin and LAMP2 (Figure 2) plus large amounts of TfR and CD147 (Figure 2). Mature donor RBC membranes, for the most part, lack these internal membranes and have only residual amounts of ER, TfR and CD147 (Figure 2). We have reported previously that residual amount of monomeric TfR (98 kDa) can be detected by immunoblotting in mature donor RBC membranes (Flatt et al., 2020). As the controls used in the immunoblots were prepared from red cell concentrates (RCC) this is unlikely to be due to residual reticulocytes (see Supplementary Figure 2). The heterozygous and homozygous HS samples were included in this study as controls because these patients had reticulocytosis (Table 1). Some of the patients studied in this report had some level of reticulocytosis (Table 1) and we needed to show that the residual reticulocyte proteins in the variant samples were not simply due to increased reticulocyte count. Interestingly, we show here that both heterozygous and homozygous HS RBCs appear to mature normally; the heterozygous HS RBC membranes have much the same protein profile as the control RBC membranes (Figure 2A and Supplementary Table 1). The homozygous HS RBC membranes do contain some residual mitochondrial proteins and increased amounts of dimeric TfR compared to the control RBC membranes (Figure 2B) but this is probably due to the trafficking defect found in these homozygous HS cells (Toye et al., 2008) or it may reflect the high (17.5%) reticulocyte count in this sample (the HS-hom. sample shows the level of these reticulocyte proteins to be expected in the RBC membrane from a sample with a high reticulocyte count). However, overall the data indicates that the RBCs (non-reticulocytes) in these HS samples mature normally although, as HS RBCs vesiculate and lose plasma membrane continually in the circulatory system, we cannot rule out the possibility that mitochondrial, lysosomal and ER membranes, CD147 and TfR have been lost in these vesicles.

**FIGURE 3:**
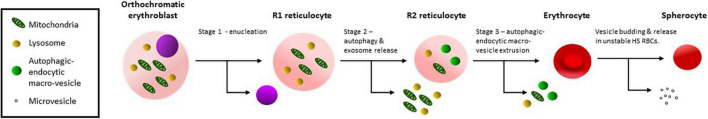
Schematic diagram of reticulocyte maturation. Stage 1: Orthochromatic erythroblasts enucleate producing an R1 reticulocyte. Stage 2: Organelles, lysosomes and obsolete cytoplasmic proteins are removed by autophagy and exosome release producing an R2 reticulocyte, probably occurs in the bone marrow. Stage 3: Residual organelles, internal membranes and obsolete membrane proteins are removed in endocytic-autophagic macro-vesicles, extruding through the cell membrane, probably involving macrophages and producing an R3 erythrocyte. Spherocytosis results from membrane budding and involves stomatin, a mechanism that is probably not involved in reticulocyte maturation.

The largest variant RBC studied was OHSt which are similar in size to nascent R1 reticulocytes (Table 1). These cells were the least mature of the variants studied and contained large amounts of mitochondrial, ER and lysosomal proteins (Figures 2A,B and Supplementary Table 1). The OHSt RBCs also contained large amounts of TfR, including the presumed dimeric form found in cRBCs, and CD147, including the unglycosylated form found in cRBCs (Figures 2A,B). Together these results suggest that the OHSt RBCs have not matured much further than the R1 stage. Next in size are the sdCHC, CHC and SAO variant RBCs (Table 1). These cells all cleared the mitochondrial and lysosomal proteins but retained more ER membranes and the SAO and CHC RBCs had slightly more CD147 than the controls (Figures 2C-E and Supplementary Table 1). The sdCHC and CHC RBC membranes contained more dimeric TfR than controls however as previously reported (Flatt et al., 2020) the SAO RBC membranes contained less TfR than the donor control RBC membranes (Figure 2C). We previously showed that SAO band 3 accumulates in SAO erythroblasts in internal vesicles disrupting cytokinesis and enucleation and blocking the intracellular milieu with vesicle aggregates that may affect the recycling of TfR (Flatt et al., 2020). Nonetheless, this group of variants, with MCVs that match the average size of an R2 reticulocyte, all matured further than the OHSt RBCs, clearing mitochondrial and lysosomal proteins. The maturation of R2 reticulocytes to mature RBCs is known to involve the final clearance of ER and other membrane fragments and excess proteins (TfR, CD147 etc.) and a final reduction in size involving the expulsion of autophagic-endocytic vesicles (Mankelow et al., 2016). This is probably followed by further dehydration and cytoskeleton rearrangement (Figure 3).

### The Role of Stomatin

Interestingly, the presence or absence of stomatin appeared to have little effect on the ability of the reticulocyte to mature. CHC and sdCHC RBCs matured to much the same level despite the near absence of stomatin in the sdCHC RBCs (Figure 2D). Stomatin is known to be involved in membrane budding as occurs in the circulation and during RBC storage (Salzer et al., 2008). Our data suggests that the budding mechanism of vesiculation is not employed in reticulocyte maturation. However, stomatin is also involved in the formation of the autophagic-endocytic vesicles produced in the final stages of reticulocyte maturation (Mankelow et al., 2016) and lack of stomatin, depending on when it is lost in these variant cells, may affect this process.

## Conclusion

We describe here a number of variant RBCs whose size appears to correlate with their level of reticulocyte maturation. The sizes of the variant cells are also similar to the sizes of reticulocytes at different stages of reticulocyte maturation. Numerous mechanisms of reticulocyte maturation have been described but it has been unclear as to whether these occur simultaneously or in stages. Our data, and that of others (Mel et al., 1977; Koury et al., 2005; Mankelow et al., 2016; Rhodes et al., 2016; Du et al., 2020; Mei et al., 2021), suggests that different mechanisms of maturation occur at different stages. Mitochondria and lysosomes predominantly being lost early probably through an autophagy and exosome release mechanism. Residual ER, TfR and CD147 predominantly lost later probably through the autophagic-endocytic macrovesicle mechanism (Figure 3). Stomatin does not appear to be required, although lack of stomatin may affect maturation in OHSt RBCs, but the membrane budding mechanism of vesiculation that occurs in RBC storage is probably not involved in reticulocyte maturation. No doubt all of these mechanisms overlap to some extent throughout the process but we believe this data provides a unique insight into the process of reticulocyte maturation and may also be useful in understanding the hemolytic anemia experienced by individuals with these conditions.

## Data Availability Statement

Publicly available datasets were analyzed in this study. This data can be found here: https://www.ncbi.nlm.nih.gov/snp/rs863225468, https://www.ncbi.nlm.nih.gov/snp/rs769664228, https://www.ncbi.nlm.nih.gov/snp/rs864309514, and https://www.ncbi.nlm.nih.gov/snp/rs863225461.

## Ethics Statement

The studies involving human participants were reviewed and approved by National Health Service National Research Ethics Service South West. The patients/participants provided their written informed consent to participate in this study.

## Author Contributions

CS-H and JF designed and performed the immunoblotting experiments. CS-H analyzed the data. SK conducted the electron microscopy experiments. LB designed the study, analyzed data, and wrote the manuscript. All authors edited the manuscript.

## Author Disclaimer

The views expressed are those of the authors and not necessarily those of the National Health Service, NIHR, or the Department of Health and Social Care.

## Conflict of Interest

The authors declare that the research was conducted in the absence of any commercial or financial relationships that could be construed as a potential conflict of interest.

## Publisher’s Note

All claims expressed in this article are solely those of the authors and do not necessarily represent those of their affiliated organizations, or those of the publisher, the editors and the reviewers. Any product that may be evaluated in this article, or claim that may be made by its manufacturer, is not guaranteed or endorsed by the publisher.
