# Supplementary material for: Reticulocyte Maturation and Variant Red Blood Cells
Source: Front Physiol. 2022 Mar 7;13:834463. doi: 10.3389/fphys.2022.834463 (PMC8959883; doi:10.3389/fphys.2022.834463)
Supplement: Supplementary file 1 [file Table_1.docx]

**Supplementary data**

**Case study – heterozygous Hereditary spherocytosis (HS) sample**

The index case (II.2) was referred to the Haematology Department, Bristol Royal Infirmary in 1995 with anaemia. Blood films showed spherocytosis (data not shown). There was a family history of hereditary spherocytosis (HS) and RBC membrane analysis showed a reduction in band 3 protein with a sharper band that migrated with faster mobility (data not shown). Blood samples were collected from all available family members (see Figure 1). DNA sequence analysis found that all family members with HS had a heterozygous g>t mutation (c.1431+1) in the donor splice site of intron 12 of *SLC4A1*. The HS-het individual (II-2) was splenectomised in December 1995.

Methods:

The buffy coats were isolated by centrifugation (1,000 *g*, 4°C, 10 min) of the whole blood samples. Genomic DNA was isolated, using a DNA extraction kit (Qiagen, UK), and the coding regions of each of exons 2 to 20 of the human *SLC4A1* gene were analysed for single-strand conformation polymorphisms (SSCP) after polymerase chain reaction (PCR) amplification of genomic DNA in the presence of ^32^P-labeled dATP (3000 Ci/mmol), using primers based on the sequences at the intron–exon junctions as described (Bruce et al., 1997). The SSCP analysis revealed an altered pattern in exon 12 and DNA sequence analysis of exon 12 found that all family members with HS had a heterozygous g>t mutation (c.1431+1) in the donor splice site of intron 12 of *SLC4A1*.

I.1

I.2

II.1

II.2

III.1

III.3

III.2

**Figure 1. Family tree**. A heterozygous HS patient (II.2) was referred for DNA analysis. DNA from her father (I.1), husband (II.1) and three children (III.1, III.2, III.3) was also analysed.

Reference:

Bruce LJ, Cope DL, Jones GK, Schofield AE, Burley M, Povey S, Unwin RJ, Wrong O, Tanner MJ. Familial distal renal tubular acidosis is associated with mutations in the red cell anion exchanger (Band 3, AE1) gene. J Clin Invest. 1997 Oct 1;100(7):1693-707.

**Methods**

**Scanning electron microscopy**

Cells were prepared for scanning electron microscopy as described in Griffiths et al., 2012a. Briefly, approximately 5-10 x10^5^ cells before or after filtration were seeded onto poly-L-lysine (Merck)–coated 13-mm cover slips and fixed with 1% (w/v) glutaraldehyde (Merck) followed by 1% (v/v) osmium tetroxide (Merck). The cells were dehydrated using a graded ethanol series and the coverslips were dried with hexamethyldisilazane (Merck) before mounting. The specimens were sputter coated using an EMITECH K575X Sputter Coater (Quorum Technologies) and imaged using a Quanta 400 scanning electron microscope (Fei) with xT microscope server software.

**Erythrocyte membrane protein analysis**

When starting with whole blood sample tubes from healthy volunteers or patient samples, the tubes were centrifuged (1000 *g*, 4°C, 10 min) and the plasma and buffy coat removed. When starting with RBC concentrate (RCC) units, a sample of RBCs was removed from the unit. RBCs, from either starting material, were washed 3 times with phosphate buffered saline (PBS; Severn Biotech Ltd, UK). RBC ghost membrane preparations were prepared according to the haemolysis method (Dodge et al., 1963) and stored at -80°C. Preparation of cRBC membranes was as above except that the haemolysis buffer included 1x protease inhibitor cocktail set V (Calbiochem, Merck) and post-haemolysis and between each wash the sample was cooled on ice for ten minutes. SDS-PAGE and Western blotting analysis were performed as described (Bruce et al., 2003). The RBC membranes were separated under reducing conditions (50 mM dithiothreitol, Sigma-Aldrich, UK) on 10% polyacrylamide gels. Blots were analysed using semi-quantitative scanning densitometry with the Kodak Gel100 system software or LI-COR Image Studio software. Densitometry analysis was carried out using Image J (v1.50i).

**Table 1 – Densitometry analysis of the immunoblotting data in Figure 2**

|  | VDAC1 | SLP2 | Calreticulin | LAMP2 | Stomatin | TfR | CD147 |
| --- | --- | --- | --- | --- | --- | --- | --- |
| *Figure 2A* | | | | | | | |
| RBC | 0.00 | 0.00 | 0.13 | 0.01 | 1.66 | 0.21 | 0.20 |
| cRBC | 1.00 | 1.00 | 1.00 | 1.00 | 1.00 | 1.00 | 1.00 |
| OHSt | 0.82 | 0.37 | 0.49 | 0.33 | 0.29 | 0.82 | 1.52 |
| HS het. | 0.00 | 0.001 | 0.16 | 0.02 | 2.74 | 0.07 | 0.40 |
| *Figure 2B* | | | | | | | |
| RBC | 0.00 | 0.00 | 0.14 | 0.08 | 0.71 | 0.27 | 0.49 |
| cRBC | 1.00 | 1.00 | 1.00 | 1.00 | 1.00 | 1.00 | 1.00 |
| OHSt | 0.94 | 0.12 | 0.56 | 0.34 | 0.14 | 0.46 | 1.11 |
| HS hom. | 0.07 | 0.10 | 0.05 | 0.00 | 0.66 | 0.37 | 0.49 |
| *Figure 2C* | | | | | | | |
| RBC | 0.01 | 0.03 | 0.09 | 0.001 | 1.41 | 0.22 | 0.34 |
| cRBC | 1.00 | 1.00 | 1.00 | 1.00 | 1.00 | 1.00 | 1.00 |
| SAO | 0.00 | 0.05 | 0.42 | 0.12 | 1.60 | 0.10 | 0.50 |
| *Figure 2D* | | | | | | | |
| RBC | 0.02 | 0.02 | 0.10 | 0.08 | 1.56 | 0.17 | 0.32 |
| cRBC | 1.00 | 1.00 | 1.00 | 1.00 | 1.00 | 1.00 | 1.00 |
| sdCHC | 0.01 | 0.02 | 0.16 | 0.06 | 0.04 | 0.23 | 0.38 |
| *Figure 2E* | | | | | | | |
| RBC | 0.00 | 0.00 | 0.20 | 0.00 | 0.76 | 0.19 | 0.02 |
| CHC | 0.00 | 0.00 | 0.37 | 0.00 | 0.76 | 0.47 | 0.27 |
| cRBC | 1.00 | 1.00 | 1.00 | 1.00 | 1.00 | 1.00 | 1.00 |

Note: The HS-het. RBC membranes analysed in Figure 2A and Suppl. Table 1 were prepared in 1996 post-splenectomy.

**A)**


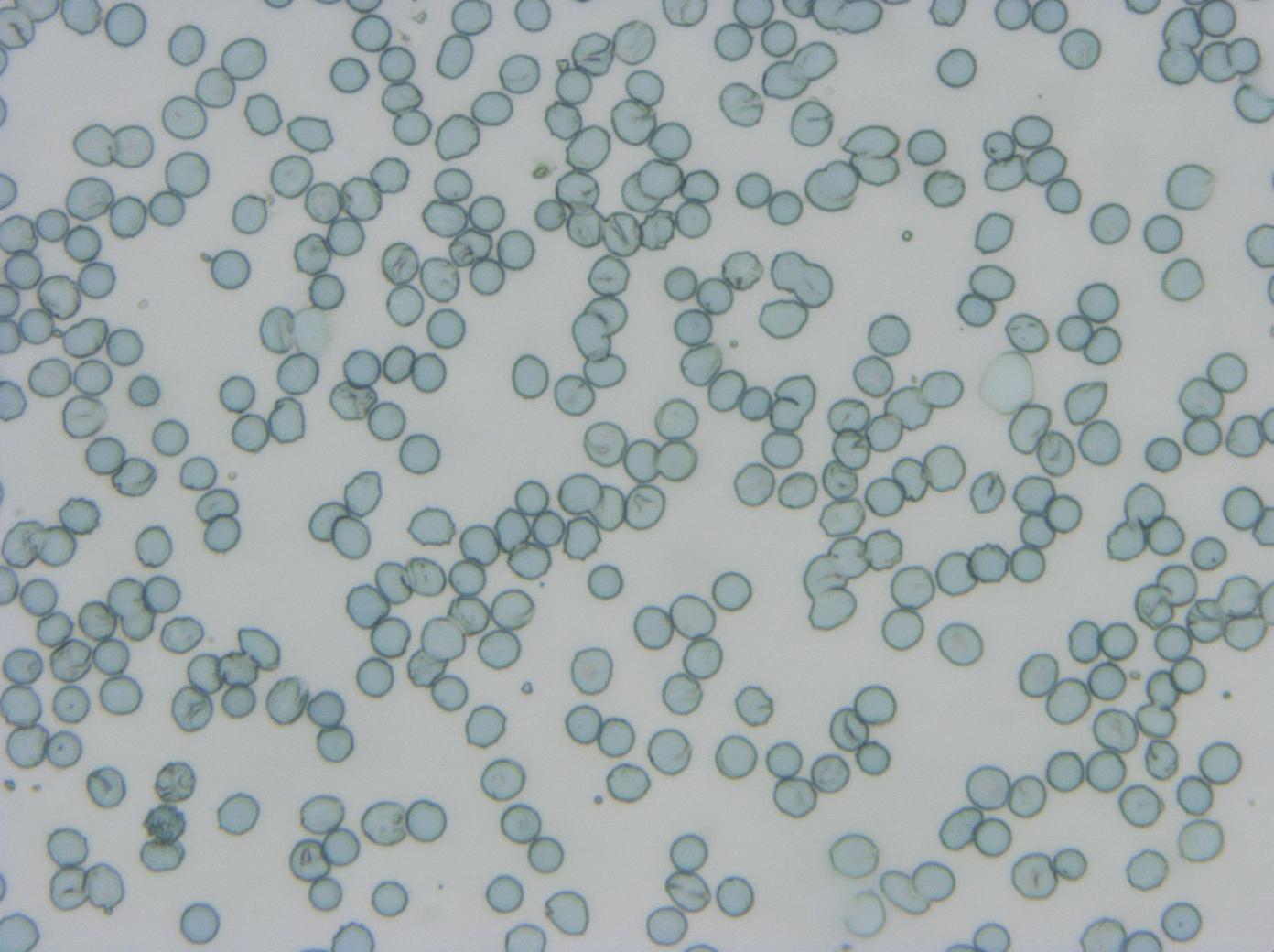


**10 μm**


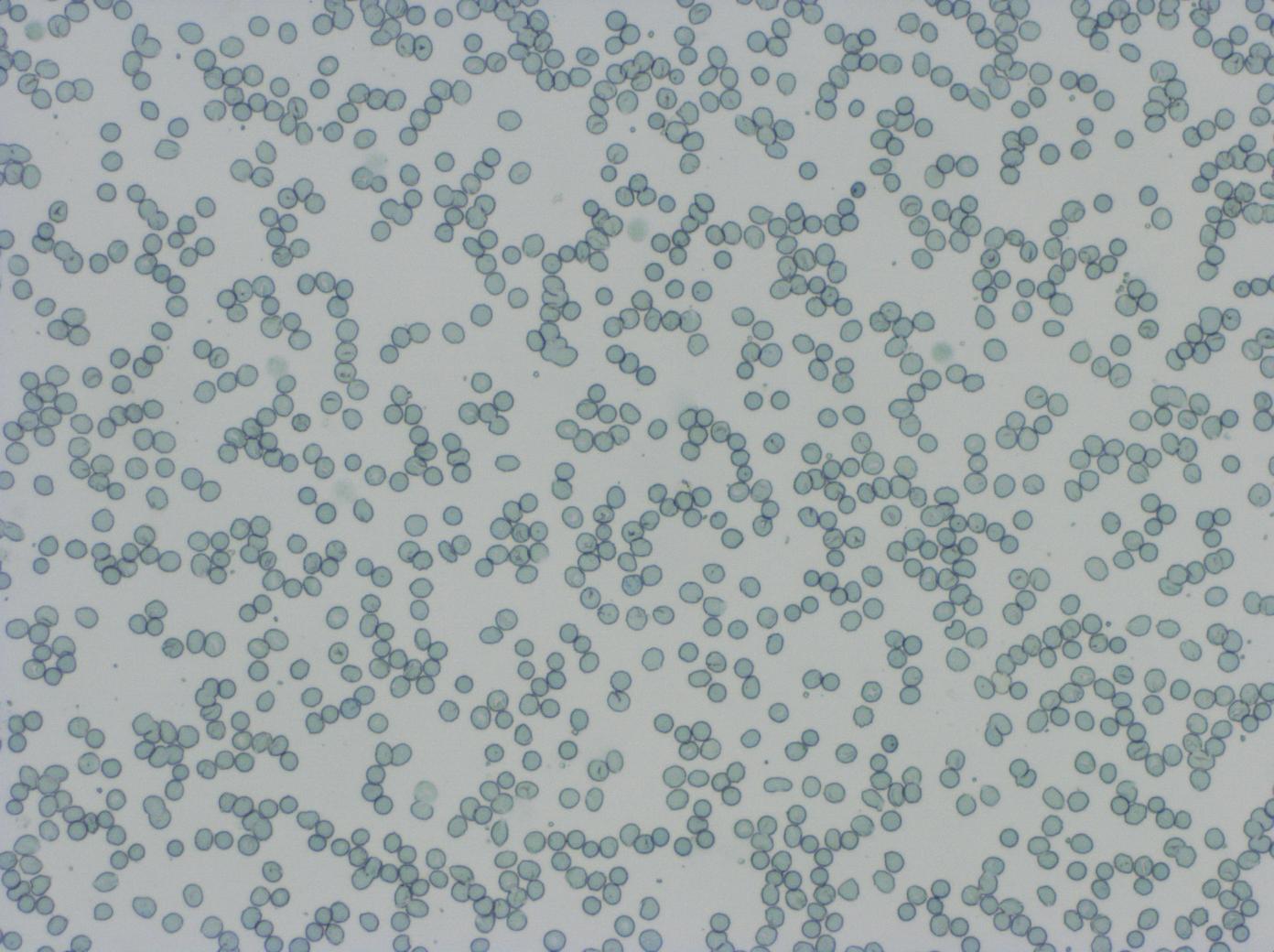


**10μm**

**B)**


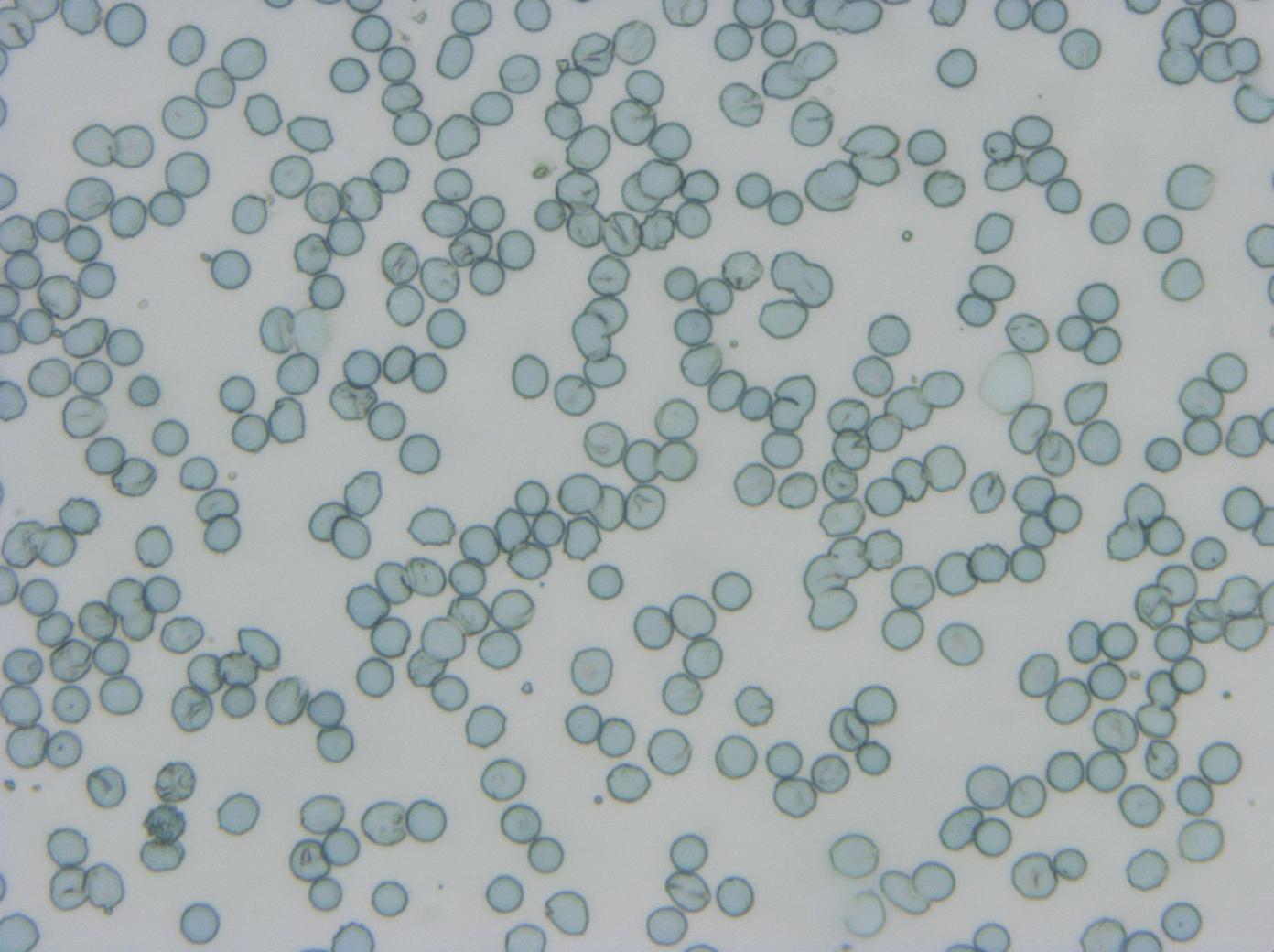


**10μm**

**Figure 2. Reticulocyte stain of a red cell concentrate unit (RCC) sample.** New methylene blue (Sigma-Aldrich #R4132) was used to identify reticulocyte content in red cell concentrate units. Three drops of RBCs were mixed with two drops of reticulocyte stain, after a 10 minute incubation at room temperature, a drop of the stained blood was placed on a microscope slide and a blood smear was prepared. Images show the complete lack of reticulocytes in red cell concentrate units. A=x40, B=x20
